# Serum 25-Hydroxyvitamin D Levels, phosphoprotein enriched in diabetes gene product (PED/PEA-15) and leptin-to-adiponectin ratio in women with PCOS

**DOI:** 10.1186/1743-7075-8-84

**Published:** 2011-11-23

**Authors:** Silvia Savastano, Rossella Valentino, Carolina Di Somma, Francesco Orio, Claudia Pivonello, Federica Passaretti, Valentina Brancato, Pietro Formisano, Annamaria Colao, Francesco Beguinot, Giovanni Tarantino

**Affiliations:** 1 Department of Molecular and Clinical Endocrinology and Oncology, Division of Endocrinology, University Federico II of Naples, Via S. Pansini 5, Naples, 80131, Italy; 2 Institute of Experimental Endocrinology and Oncology-CNR, Via S. Pansini 5, Naples, 80131, Italy; 3 IRCCS SDN Foundation, Via Gianturco 113, Naples, 80143, Italy; 4 Department of Endocrinology, University Parthenope of Naples, Via Amm. F. Acton 38, Naples, 80133, Italy; 5 Department of Cellular and Molecular Biology and Pathology, University Federico II of Naples, Via S. Pansini 5, Naples, 80131, Italy; 6 Department of Clinical and Experimental Medicine, University Federico II of Naples, Via S. Pansini 5, Naples, 80131, Italy

**Keywords:** 25-hydroxyvitamin D, PED/PEA-15, Leptin-to-adiponectin ratio, PCOS, apoptosis

## Abstract

**Background:**

Polycystic ovary syndrome (PCOS) is frequently associated with hypovitaminosis D. Vitamin D is endowed with pleiotropic effects, including insulin resistance (IR) and apoptotic pathway. Disruption of the complex mechanism that regulated ovarian apoptosis has been reported in PCOS. Phosphoprotein enriched in diabetes gene product (PED/PEA-15), an anti-apoptotic protein involved in type 2 diabetes mellitus (T2DM), is overexpressed in PCOS women, independently of obesity. Leptin-to-adiponectin ratio (L/A) is a biomarker of IR and low-grade inflammation in PCOS. The aim of the study was to investigate the levels of 25-hydroxy vitamin D (25(OH)D), and L/A, in association with PED/PEA-15 protein abundance, in both lean and overweight/obese (o/o) women with PCOS.

**Patients and Methods:**

PED/PEA-15 protein abundance and circulating levels of 25(OH)D, L/A, sex hormone-binding globulin, and testosterone were evaluated in 90 untreated PCOS patients (25 ± 4 yrs; range 18-34) and 40 healthy controls age and BMI comparable, from the same geographical area. FAI (free androgen index) and the homeostasis model assessment of insulin resistance (HoMA-IR) index were calculated.

**Results:**

In o/o PCOS, 25(OH)D levels were significantly lower, and L/A values were significantly higher than in lean PCOS (p < 0.001), while there were no differences in PED/PEA-15 protein abundance. An inverse correlation was observed between 25(OH)D and BMI, PED/PEA-15 protein abundance, insulin, HoMA-IR, FAI (p < 0.001), and L/A (p < 0.05). At the multivariate analysis, in o/o PCOS L/A, insulin and 25(OH)D were the major determinant of PED/PEA-15 protein abundance (β = 0.45, β = 0.41, and β = -0.25, respectively).

**Conclusions:**

Lower 25(OH)D and higher L/A were associated to PED/PEA-15 protein abundance in PCOS, suggesting their involvement in the ovarian imbalance between pro-and anti-apoptotic mechanisms, with high L/A and insulin and low 25(OH)D levels as the main determinants of PED/PEA-15 protein variability. Further studies, involving also different apoptotic pathways or inflammatory cytokines and granulosa cells are mandatory to better define the possible bidirectional relationships between 25(OH)D, PED/PEA-15 protein abundance, leptin and adiponectin in PCOS pathogenesis.

## Background

Obesity, predominantly intra-abdominal visceral adipose tissue, is observed in 30%-75% of women with polycystic ovary syndrome (PCOS) [1], with hyperandrogenism and insulin resistance (IR) as other common features of this syndrome [2]. Obesity is considered to be a risk factor for hypovitaminosis D [3]. A number of cross-sectional studies have evidenced the association between obesity, hypovitaminosis D and PCOS cohorts [4-8], although hypovitaminosis D have been considered as the consequence of obesity and IR *per se *[7].

Vitamin D is endowed with pleiotropic effects on a wide spectrum of intracellular regulatory mechanisms, including insulin metabolism [9], or intrinsic apoptotic pathway, on both classical and nonclassical tissues, such as ovary [10]. Dysregulation of the complex mechanism that regulated ovarian apoptosis has been reported in PCOS [11].

Phosphoprotein enriched in diabetes gene product (PED/PEA-15) is an anti-apoptotic protein involved in IR and type 2 diabetes mellitus (T2DM) [12]. We previously reported that PED/PEA-15 is overexpressed in T2DM patients [13] and in PCOS women [14]. In both groups this association was independent of obesity, thus suggesting that PED/PEA-15 overexpression might represent a T2DM or PCOS specific feature, likely linked to IR, although also different pathogenetic mechanisms influencing PED/PEA -15 expression in PCOS, such as apoptosis, have not been excluded.

Circulating levels of leptin and adiponectin, two adipose tissue-derived hormones with opposing associations with the metabolic syndrome (MetS) and coronary heart disease, are altered in PCOS [15], thus contributing through the low-grade chronic inflammation to the long-term metabolic consequence of the syndrome [16], and possibly, to the dysregulation between apoptotic and antiapoptotic mechanisms [17]. Finally, the ratio of leptin-to-adiponectin (L/A), has been reported to represent a better marker for obesity, IR, and MetS than each single adipokine [18], in particular in female population [19]. Although there is still some debate, a number of recent studies supported a role for L/A as a biomarker of both IR and low-grade inflammation also in women with PCOS [20].

Taking into account their involvement in obesity and apoptosis, the aim of our study was to investigate the balance between circulating levels of 25-hydroxy vitamin D (25(OH)D), leptin, and adiponectin, and PED/PEA-15 protein abundance, in both lean and overweight/obese (o/o) women with PCOS.

## Materials and methods

### Study design

This is an observational clinical study based on the evaluation of 25(OH)D, leptin, and adiponectin in both lean and overweight/obese (o/o) women with PCOS. The procedures used were in accordance with the guidelines of the Helsinki Declaration on human experimentation. The study was conducted without support from the pharmaceutical industry, after approval by the institutional review board of the University of Naples, Italy. The purpose of the protocol was explained to both the patients and the controls, and written consent was obtained at the beginning of the study.

### Subjects

Ninety untreated PCOS patients (25 ± 4 yrs; range 18-34) were consecutively admitted to the Endocrinology Unit of the Department of Molecular and Clinical Endocrinology and Oncology of the Federico II University of Naples (Italy), and enrolled in this observational clinical study according to following inclusion criteria: pre-menopausal women, with a strict age range (20-40 yr) with diagnosis of PCOS; anovulatory oligo-amenorrhea; comparable clinical/biochemical hyperandrogenism; caucasian ethnicity. All subjects included in the study resided in the Naples metropolitan area (latitude 40° 49' N; elevation 17 m) and were evaluated from March trough June 2011.

To further minimize subjects variability, the presence of T2DM or abnormal glucose tolerance was excluded by the oral glucose tolerance test (OGTT). Other exclusion criteria for all subjects were: smoking or alcohol consumption, pregnancy, hypothyroidism, hyperprolactinemia, Cushing's syndrome, non-classical congenital adrenal hyperplasia; previous (within the last 6 months) use of oral contraceptives, glucocorticoids, anti-androgens, ovulation induction agents, anti-obesity drugs, or other hormonal drugs. None of the subjects was affected by any neoplastic, metabolic, hepatic, and cardiovascular disorder or other concurrent medical illness (i.e. renal disease, and malabsorptive disorders). Moreover, those with acute inflammations based on medical history, physical examination, and routine laboratory tests, including measurement of body temperature, white blood cell count (WBC) and urinalysis, were excluded. The diagnosis of PCOS was made according to the diagnostic criteria for PCOS [21]. All PCOS women enrolled in the study had clinical and/or biochemical hyperandrogenism with IR. Forty young women, among clerks, and medical and paramedical personnel of the Department of Molecular and Clinical Endocrinology and Oncology of the University "Federico II" of Naples, age and BMI comparable with the patients, from the same geographical area, with regular menstrual cycles (defined as 26-32 days in length), agreed to participate in this study and were used as controls. Exclusion criteria for controls were the same of the patients. All participants gave their informed consent before enrolment.

### Methods

The ovulatory state was investigated by pelvic or transvaginal ultrasonography (TV-USG) and plasma progesterone (P) levels. Both procedures were performed during the luteal phase of the menstrual cycle (7 days before the expected menses). The presence of fluid in the cul-de-sac at pelvic or TV-USG and a plasma P assay >32 nmol/l (>10 ng/ml) were considered to be the criteria to show that ovulation had occurred [22]. The controls were not genetically related to the PCOS group and without family history of diabetes.

Hirsutism was assessed using the Ferriman-Gallwey (FG)-score, with a score >8 indicative of hyperandrogenism [23]. A progesterone challenge test (100 mg natural progesterone im) was performed, which induced uterine bleeding in all PCOS women. The normal glucose response to the OGTT was defined according to the "Report of the Expert Committee on the diagnosis and classification of diabetes mellitus" [24].

All anthropometric measurements were taken with subjects wearing only light clothes and without shoes. In each woman, weight and height were measured to calculate the BMI [weight (kg) divided by height squared (m^2^), kg/m^2^]. Height was measured to the nearest cm using a wall-mounted stadiometer. Body weight was determined to the nearest 50 g using a calibrated balance beam scale. The degree of normal weight, overweight, or obesity was established on the basis of BMI cut-off points of 18-24.9 (lean subjects), 25-29.9, 30-34.9, 35-39.9 and > 40 kg/m^2^, respectively (o/o).

### Assays

Blood samples, obtained between 08:00 h and 09:00 h from an ante-cubital vein after an overnight fast, with the patient in the resting position, were promptly centrifuged, and serum separated and stored at -20°C until assay. The OGTT was performed using 75 g dextrose. Blood samples were obtained at 0, 30, 60, 90, 120 min for plasma glucose and insulin measurements. Fasting plasma glucose (FPG) levels were determined by the glucose oxidase method immediately after the OGTT. Circulating levels of 25(OH)D (LIAISON; DiaSorin, Saluggia (VC), Italy), fasting plasma insulin (FPI), sex hormone-binding globulin (SHBG), and testosterone (T) levels (Immulite, Diagnostic Products Co, Los Angeles, CA; n.v. 0.2-1.2 nmol/l) were measured by solid-phase chemiluminescent enzyme immunoassays. The intra-assay coefficients (CV) of variations were less than 5·5% for the 25(OH)D, insulin, and SHBG assays, and 10% for total T assay. FAI (free androgen index) was calculated as an expression of peripheral androgen activity and estimated as total T and SHBG serum concentration, according to the formula FAI = T (nmol/l)/SHBG (nmol/l) × 100. Leptin and total adiponectin (low, middle, and high molecular weight) levels were determined with commercially available enzyme-linked immunosorbent assay (ELISA) kits (AviBion: Orgenium Laboratories, Helsinki, Finland), with sensitivity 1 and 3 ng/ml, respectively; intra- and inter- assay CVs were less than 10% and 12%, respectively, for adiponectin and leptin. Vit D deficiency and insufficiency were defined as 10-30 and <10 ng/ml, respectively. The homeostasis model assessment of insulin resistance (HoMA-IR) index was calculated from FPG and FPI according to the report by the formula [FPI (μU/ml) × FPG (mmol/l)]/22.5] [25]. As a stringent measure of IR, a value of HoMA-IR > 2.0 was set (deriver by the mean ± 2 SD of our lean population), in accordance with a previuos cut-off [26].

PED/PEA-15 protein levels were measured in WBC lysates obtained from 10 to 12 ml of freshly collected uncoagulated whole blood, after separation with dextran 6%, using Western blot analysis, as previously reported [13]. For Western blot analysis WBC were solubilized at 4 C in TAT buffer, centrifuged at 500 g for 20 min, and supernatant fractions were stored at -20°C until used. The amount of 50 μg of lysate proteins were heated at 100°C in Laemmli buffer. Proteins were separated by 15% SDS-PAGE and then transferred to 0·45 mm Immobilon-P membranes (Millipore, Bedfort, MA). Filters were probed with PED/PEA-15 antiserum at 1:2000 dilution, revealed by enhanced chemiluminescence and autoradiography. The protein bands were quantified by laser densitometry and expressed as arbitrary units. As reported previously [13], PED/PEA-15 protein levels in WBC detected by Western blot analysis, correlated strongly with mRNA detected by real time RT-PCR (*p *< 0.001). In addition, PED/PEA-15 protein stability was also assessed by repeated testing, at 2, 4, and 6 months after storage, displaying intra- and inter-assay coefficients of variation <8 and <15%, respectively.

### Statistical analysis

All results are expressed as mean ± SD. Fasting 25(OH)D, insulin, PED/PEA-15 protein abundance, leptin, and adiponectin values were not normally distributed and have been logarithmically transformed. Differences between lean and overweight/obese subjects in both PCOS and Control women were analysed by unpaired *t *test. Bivariate correlations between variables were examined using Pearson's correlation coefficient. Only variables significant on univariate analysis were included in the multivariate analysis. Three multiple linear regression analysis (stepwise model, *p *to include <0.005, *p *to remove >0.1, maxstep 15), were calculated with PED/PEA-15, as dependent variable and BMI, insulin, 25(OH)D, L/A and FAI as independent variables considering PCOS women as a whole, or according to BMI (lean and o/o). To avoid multicollinearity, variables with a tolerance of 0.2 were excluded. Values ≤5% were considered statistically significant. Data were stored and analysed using the Statistical Package for Social Science program (SPSS, release 13·01, Chicago, IL).\\

## Results and Discussion

Metabolic and hormonal characteristics of the study population were reported in Table 1. Testosterone levels and FG score in PCOS women were 2.8 ± 1.1 nmol/l and 16 ± 6, respectively. Grouping PCOS women according to BMI, there were 42 lean and 48 o/o subjects. Subgroup analysis of o/o and lean women revealed differences between groups. In particular, apart from the expected differences in insulin, HoMA-IR, and androgens, 25(OH)D and adiponectin levels were significantly lower, while leptin levels and L/A values were significantly higher in o/o PCOS compared with lean PCOS. No differences in PED/PEA-15 protein abundance were evident between lean and o/o PCOS women.

**Table 1:** Metabolic and endocrine characteristics of the study population grouped according to BMI.

	PCOS women (90 subjects)			Controls (40 subjects)		
	**Lean** **(42)**	**Overweight/Obese** **(48)**	**p**	**Lean** **(20)**	**Overweight/Obese** **(20)**	**p**

Age (yrs)	24.1 ± 4.6	24.8 ± 4.0	NS	23.9 ± 3.6	25.4 ± 4.6	NS
BMI	22.1 ± 2.6	33.1 ± 5.8	<0.001	22.1 ± 1.6	34.9 ± 4.1	<0.001
25(OH)D (ng/ml)	15.1 ± 7.6	11.1 ± 5.8	0.021	48.9 ± 11.3	10.2 ± 4.7	<0.001
PED/PEA-15	290 ± 102	295 ± 113	0.745	153 ± 29	177 ± 47	0.092
Leptin (ng/ml)	25.8 ± 6.6	40.3 ± 9.1	<0.001	9.9 ± 4.8	29.4 ± 6.6	0.003
Adiponectin (ng/ml)	13.2 ± 5.7	9.4 ± 6.3	<0.001	17.4 ± 3	11.6 ± 2.5	<0.001
Insulin (μUI/ml)	11.9 ± 4.6	17.1 ± 9.4	0.014	7.6 ± 1.9	11.5 ± 2.0	<0.001
Leptin-to-adiponectin ratio	2.5 ± 1.8	5.8 ± 2.7	<0.001	0.6 ± 0.4	2.0 ± 1.7	<0.001
HoMA-IR	2.4 ± 1	3.8 ± 2.2	<0.001	1.4 ± 0.3	2.2 ± 0.3	<0.001
SHBG (nmol/l)	45.7 ± 7.2	41.3 ± 6.9	0.001	69.5 ± 3.1	63.3 ± 10.5	0.016
FAI	4.6 ± 0.8	8.7 ± 3.8	<0.001	0.8 ± 0.2	0.9 ± 0.2	0.05

Data are reported as mean ± SD. *BMI*, body mass index; *25(OH)D*, 25(OH) Vitamin D; *PED/PEA-15*, PED/PEA-15 abundance; *HoMA-IR*, homeostasis model assessment of insulin resistance; *SHBG*, sex hormone binding globulin; *FAI*, free androgen index; *FG*, Ferriman-Gallwey. PED/PEA-15 protein levels are expressed as arbitrary units. Fasting 25(OH)D, insulin, PED/PEA-15 protein abundance, leptin, and adiponectin values were not normally distributed and have been logarithmically transformed. Differences between lean and overweight/obese subjects in both PCOS and Control women were analysed by unpaired *t *test. Controls were age and BMI comparable.

**Figure 1:**
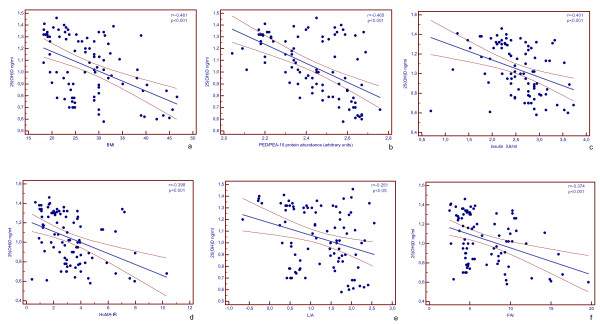
**Correlations between variables in the study population**. *25(OH)D*, 25(OH) Vitamin D; *BMI*, body mass index; *HoMA-IR*, homeostasis model assessment of insulin resistance; *FAI*, free androgen index; *L/A*, leptin-to-adiponectin ratio. Fasting 25(OH)D, insulin, PED/PEA-15 protein abundance, leptin, and adiponectin values were not normally distributed and have been logarithmically transformed. PED/PEA-15 protein bands were quantified by laser densitometry and expressed as arbitrary units [13]. Bivariate correlations between variables were examined using Pearson's correlation coefficient and their values are singularly evidenced.

Correlations between variables in the study population were reported in Figure 1. In PCOS women, an inverse correlation was observed between 25(OH)D and BMI (a), PED/PEA-15 protein abundance (b), insulin (c), HoMA-IR (d), and FAI (e) (r= -0.474, -0.553, -0.380, -0.407, -0.374, respectively; p < 0.001), and L/A (f) (r= -0.306, p < 0.05). At the multivariate analysis, with PED/PEA-15 protein abundance as dependent variable only insulin and 25(OH)D remained in the model (β = 0.39 and β = -0.32, respectively) (Table 2). The results of the analysis were different, however, in the two group of PCOS women, as the major determinants of PED/PEA-15 protein abundance were BMI and insulin in lean PCOS (β = 0.65 and β = 0.26, respectively) (Table 3), and L/A, insulin and 25(OH)D in o/o PCOS (β = 0.45, β = 0.41 and β = -0.25, respectively) (Table 4).

**Table 2:** Multiple linear regression analysis in PCOS women.

Independent variables: BMI, insulin, 25(OH)D, L/A, FAI			
Step	Variables inserted in the model	p	R^2^
1	insulin	<0.001	0.26
2	insulin 25(OH)D	<0.001 0.001	0.33

Variables excluded from the model: BMI, L/A, FAI			

Variations in PED/PEA-15 abundance explained by stepwise model (*p *to include < 0.05, *p *to remove > 0.1), with selected variables in PCOS women (n = 90). *BMI*, body mass index; *25(OH)D*, 25(OH) Vitamin D; *PED/PEA-15*, *L/A*, leptin-to-adiponectin ratio; *FAI*, free androgen index.

**Table 3:** Multiple linear regression analysis in lean PCOS women.

Independent variables: BMI, insulin, 25(OH)D, L/A, FAI			
Step	Variables inserted in the model	p	R^2^
1	BMI	<0.001	0.52
2	BMI insulin	<0.001 0.021	0.58

Variables excluded from the model: 25(OH)D, L/A, FAI			

Variations in PED/PEA-15 abundance explained by stepwise model (*p *to include < 0.05, *p *to remove > 0.1) in lean PCOS women (n = 42). *BMI*, body mass index; *25(OH)D*, 25(OH) Vitamin D; *PED/PEA-15*, *L/A*, leptin-to-adiponectin ratio; *FAI*, free androgen index.

**Table 4:** Multiple linear regression analysis in overweight/obese PCOS women.

Independent variables: BMI, insulin, 25(OH)D, L/A, FAI			
Step	Variables inserted in the model	p	R^2^
1	L/A	<0.001	0.36
2	L/A Insulin	<0.001 <0.001	0.58
3	L/A Insulin 25(OH)D	<0.001 <0.001 0.015	0.63

Variables excluded from the model: BMI, FAI			

Variations in PED/PEA-15 abundance explained by stepwise model (*p *to include < 0.05, *p *to remove > 0.1) with selected variables in o/o PCOS women (n = 48). *BMI*, body mass index; *25(OH)D*, 25(OH) Vitamin D; *PED/PEA-15*, *L/A*, leptin-to-adiponectin ratio; *FAI*, free androgen index.

Our data evidenced that in women with PCOS low levels of 25(OH)D and PED/PEA-15 protein abundance are associated to high insulin, HoMA-IR, and L/A values. According to previous data hypovitaminosis D was evident in all PCOS women, with 25(OH)D levels being significantly lower in o/o than in lean PCOS women. Insulin was the major determinant of PED/PEA-15 protein abundance, explaining about 26% of PED/PEA-15 protein abundance variability, while 25(OH)D levels added another 7% of its variability. In that our data confirmed the association between insulin and 25(OH)D [7], but evidenced also a novel association between 25(OH)D, PED/PEA-15 protein abundance, and L/A in PCOS women.

A number of cross-sectional studies have evidenced the association between hypovitaminosis D and PCOS cohorts [4-8], although low serum 25(OH)D concentrations have been considered as the consequence of obesity and IR *per se *[5-7]. Also an altered L/A has been already reported in PCOS women, as a biomarker of both IR and low-grade inflammation [15,17,27,28]. However, to the best of our knowledge, all these variables in PCOS women have been investigated in the setting of IR or MS, while it is well-known that either 25(OH)D or low-grade inflammation display strict relationships with apoptotic and antiapoptotic mechanisms [29]. Thus, the association of 25(OH)D and L/A ratio with the anti-apoptotic protein PED/PEA-15 might suggest a different scenario in PCOS women.

Loss of the ovarian apoptotic mechanism has been reported to account PCOS appearance [11,30,31], with hyperandrogenism as one of the proposed mechanisms affecting the balance between the ovarian expression of pro and anti-apoptotic proteins [32]. In particular, follicular atresia is associated with an imbalance in the antiapoptotic effect of the Bcl-2 family members [33], while gonadotropin treatment inhibits granulosa cell apoptosis and follicular atresia through the reduction of the expression of the proapoptotic protein Bax [32]. Recent studies have revealed that Vit D is involved in the control of various cellular processes, including apoptosis, *via *Bcl-family up-regulation and Bax down-regulation, but the mechanisms underlying this action have not been fully explored [10,34]. Similarly, leptin and adiponectin exherted opposite effects also in the complex control of apoptosis-antiapoptosis mechanisms. In particular, leptin acts as a mitogenic factor in a variety of cell types, including ovary [35], where it has been found to interfere with ovulation rate [36]. On the other hand, adiponectin, whose receptors are expressed in ovary in human [37], has been recently reported to activate the Mitogen-Activated Protein Kinase (MAPK) cascade in granulosa cells, in the context of the classical adiponectin anti-apoptotic effects [37-39].

In that, our data evidenced that, hypovitaminosis D and impaired L/A played an adjunctive role to insulin in influencing the increase in the anti-apoptotic protein PED/PEA-15 abundance and, therefore, contributing to alter the equilibrium between anti and pro-apoptotic factors in PCOS.

Of interest, grouping PCOS women according to BMI there were different associations between the study variables. In particular, in lean PCOS BMI explained 52% of PED/PEA-15 protein abundance variability, and insulin added only another 6% of its variability; in o/o PCOS women L/A, insulin and 25(OH)D explained 37%, 22% and 5% of PED/PEA-15 protein abundance variability, respectively. Thus, it is tempting to speculate that in PCOS women, along with the progressive increase in BMI, A/L and IR induced by increased amount of dysfunctional adipocytes are involved in the higher activation of anti-apoptotic pathways, such as PED/PEA-15 protein.

The alteration of the dynamics of the loss of preantral follicle by atresia might contribute to increase androgen secretion from atresic follicles, that on turn, resulted not only in further increase in follicle atresia [32], but also in IR and PED/PEA-15 protein abundance. In line with hypothesis, we found opposite correlations between 25(OH)D, PED/PEA-15 protein and L/A with FAI, the main biochemical endocrine PCOS features. In particular, 25(OH)D exhibited an inverse, while PED/PEA-15 protein and L/A showed a positive correlation with FAI. Apart from a clear effect of gender differences [40], the inverse association of 25(OH)D with testosterone and FAI in the setting of PCOS, despite some controversies [8], and the positive association of PED/PEA-15 protein abundance with hyperandrogenism, were according to previously published data [14], further suggesting the involvement of bidirectional influences between both variables in the progression of the disease.

Due to the cross-sectional design of the study, these results must be regarded as preliminary data and cannot be generalized beyond the cases studied, nor we can draw conclusions on the natural progression of these relationships over time. Moreover, we evaluated PED/PEA-15 protein abundance in WBC and not in ovary cells; thus, although we previously reported that PED/PEA-15 protein levels in WBC correlated strongly with mRNA detected by real time RT-PCR PED/PEA- 15 protein amount in fat and in skeletal muscle tissues [13], we need to be aware of possible unexplored differences between WBC and ovary cell PED/PEA-15 protein expression. Taking into account that laparoscopy is not routinely performed in PCOS for ethical considerations, we did not obtain granulosa cells preparations from women with PCOS in this preliminary investigation.

## Conclusions

Although further studies are mandatory, involving also different apoptotic pathways or inflammatory cytokines and PCOS granulosa cells, the correlations between 25(OH)D, PED/PEA-15 protein abundance, L/A ratio, and hyperandrogenism in our sample of PCOS women, with high insulin and low 25(OH)D levels as the main determinants of PED/PEA-15 protein abundance, suggested the involvement of all these variables in the imbalance between pro-and anti-apoptotic factors responsible for the increased follicular atresia in PCOS.

## Abbreviations

*PCOS*: polycystic ovary syndrome; *25(OHD)*: 25-hydroxy vitamin D; *IR*: insulin resistance; *PED/PEA-15*: phosphoprotein enriched in diabetes gene product; *T2DM*: type 2 diabetes mellitus; *MetS*: metabolic syndrome; *L/A*: leptin-to-adiponectin ratio; *TV-USG*: transvaginal ultrasonography; *P*: plasma progesterone; *FG score*: Ferriman-Gallwey score; *BMI*: body mass index; *OGTT*: oral glucose tolerance test; *FPG*: fasting plasma glucose; *FPI*: fasting plasma insulin; *SHBG*: sex hormone-binding globulin; *T*: testosterone; *FAI*: free androgen index.

## Competing interests

The authors declare that they have no competing interests.

## Authors' contributions

SS and RV are first authors of the manuscript as they equally contributed to the study, participated in its design and coordination, and helped to draft the manuscript. FO and CDS performed the clinical investigation. BV, CP, FP, and FP gathered the data. CA and BF critically revised the manuscript. TG contributed to the study design, to performing statistics, and drafting manuscript. All Authors read and approved the final manuscript.
